# [Corrigendum] Knockdown of circ_0067934 inhibits gastric cancer cell proliferation, migration and invasion via the miR‑1301‑3p/KIF23 axis

**DOI:** 10.3892/mmr.2024.13307

**Published:** 2024-08-14

**Authors:** Jin Xu, Nan Sang, Junning Zhao, Wei He, Nannan Zhang, Xueliang Li

Mol Med Rep 25: 202, 2022; DOI: 10.3892/mmr.2022.12718

Following the publication of the above article, an interested reader drew to our attention the fact that the forward primer reported in Table I on p. 3 for miR-545-3p (5’-TGGCTCAGTTCAGCAGGAAC-3’) was actually for miR-24-3p (5’-UGGCUCAGUUCAGCAGGAACAG-3’). Upon performing an independent analysis of the primer sequences in the Editorial Office, the sequence presented for miR-670-5p also appeared to have potentially been written incorrectly. After having drawn these matters to the attention of the authors, they realized that these sequences had indeed been written incorrectly in Table I.

The corrected version of Table I, featuring the correct forward and reverse primer sequences for both miR-670-5p and miR-545-3p, is shown opposite. The authors wish to thank the interested reader for drawing this error to their attention, and are grateful to the Editor of *Molecular Medicine Reports* for allowing them this opportunity to publish a Corrigendum. They also apologize to the readership for any inconvenience caused.

## Figures and Tables

**Table I.** Sequences of primers for reverse transcription-quantitative PCR.

Name	Primer	Sequence (5′-3′)
circ_0067934	F:	5’-TAGCAGTTCCCCAATCCTTG-3’
	R:	5’-CACAAATTCCCATCATTCCC-3’
miR-5047	F:	5’-GCCTAGACGAGACACAGTGC-3’
	R:	5’-GCCAAGACCTTACAACCGCA-3’
miR-1301-3p	F:	5’-GCCCGCTTGCAGCTGCCTGGGAG-3’
	R:	5’-GTGCAGGGTCCGAGGT-3’
miR-670-5p	F:	5’-GCCGAGGTCCCTGAGTGTAT-3’
	R:	5’-CTCAACTGGTGTCGTGGA-3’
miR-345-3p	F:	5’-GCCGAGAGGGGTCTGGAGA-3’
	R:	5’-CTCAACTGGTGTCGTGGA-3’
miR-545-3p	F:	5’-TCGGCAGGTCAGCAAACATTTA-3’
	R:	5’-CTCAACTGGTGTCGTGGA-3’
KIF23	F:	5’-AGACAGAAGGCGAGGGATG-3’
	R:	5’-GGAGACGAATTGGTGGTGC-3’
U6	F:	5’-CTCGCTTCGGCAGCACATATA-3’
	R:	5’-ACGCTTCACGAATTTGAGTGTC-3’
GAPDH	F:	5’-GTCAAGGCTGAGAACGGGAA-3’
	R:	5’-AAATGAGCCCCAGCCTTCTC-3’

miR, microRNA; circ, circular RNA; KIF23, kinesin family member 23; F, forward; R, reverse.

